# Fertility preservation in the pediatric population—experience from a German Cryobank for ovarian tissue

**DOI:** 10.3389/fendo.2022.995172

**Published:** 2022-11-10

**Authors:** Dunja M. Baston-Büst, Alexandra P. Bielfeld

**Affiliations:** Department of OB/GYN and REI, UniKiD/UniCareD, Medical Hospital University of Düsseldorf, Düsseldorf, Germany

**Keywords:** cryopreservation, freezing, juvenile, children, infertility

## Abstract

Counseling children on the possibility of fertility preservation prior to a gonadotoxic treatment supports the decision-making process, taking into account that the patients are in a very vulnerable and mentally exhausting situation following the diagnosis. Referral to specialists can be optimized on-site by routing slips with contact addresses, phone numbers, and mail contacts; available time slots for consultation; possibly offers for cost coverage; and an easy-to-understand information leaflet about the different options available. Some of the options for fertility preservation in the prepubertal population especially are still experimental. The unique possibility of fertility preservation before the onset of the gonadotoxic therapy, which may cause premature ovarian insufficiency or azoospermia in the future, should be highlighted.

## Introduction

### Cancer registries

The cases of children suffering from pediatric cancer pathologies are listed in cancer registries worldwide. The first challenge is that the registers are not able to represent all cases; e.g., the German register for pediatric cancer lists an estimated 95% of all cases (https://www.kinderkrebsregister.de/dkkr/ueber-uns/uebersicht.html). From the beginning of data acquisition in the 1980s in Germany, when the registry was founded in a University setting, a worldwide connection (e.g., the International Association of Cancer Registries or the European Network of Cancer Registries) was pursued to present a comprehensive data set. A further challenge is that the data acquisition ends at the patient age of 18.

## Childhood cancer and infertility

Due to the aforementioned limitations in data collection, patient follow-up is proving difficult. For example, cases of premature ovarian failure (POF) in adolescent female cancer survivors or azoospermia in adolescent male cancer survivors cannot be linked to the type of cancer or the treatment that was received during childhood. Thirteen out of 30 female patients counseled in our center for reproductive endocrinology and infertility (UniKiD) due to POF are former pediatric cancer treatment patients. These female patients reported the cancer treatment they received during childhood in the medical history questionnaire. Most of these patients did not suspect that the POF resulted from the treatment of their childhood cancer. A recent study on the use of ART by childhood cancer survivors highlights the higher incidence of ART treatments compared with general ART statistics in Germany. Fresh cycle oocytes or sperm were mainly used during ART treatment (1). There were more multiples born and a higher prevalence for low birth weight in the offspring of cancer survivors, whereas the prevalence for preterm birth or small for gestational age were comparable to spontaneously conceived offspring of cancer survivors. Neither childhood cancer nor congenital malformations were found to be increased in the offspring (1). Therefore, it is extraordinarily important that every patient of reproductive age and every child should have the possibility to be counseled in a specialized center by gynecologists, reproductive/endocrine specialists, or urologists/andrologists before the gonadotoxic treatment starts as recommended by the current S2k guideline on fertility preservation for patients with malignant diseases (2). During counseling, the risks of infertility as a result of the gonadotoxic treatment (chemotherapy or surgery or radiation), the possibilities for fertility preservation, the risk of metastasis for systemic diseases, and the individual case should be discussed (3). There should also be a follow-up during puberty or if the onset of puberty takes place after the age of 14 with hormone analyses, ejaculate analyses, and Tanner scoring as recommended by endocrinologists. All data of primary or secondary amenorrhea or azoospermia should be sent to the registries, keeping in mind the lack of registries after the age of 18 (2).

## Options of fertility preservation in the prepubertal child

Fertility preservation in the young child is limited due to the outstanding puberty and physical immaturity. The freezing of ovarian tissue biopsies is the only option for young girls and the only promising option for females if less than 14 days remain until the beginning of the gonadotoxic therapy (4, 5). Concerning young boys, the techniques are even still more experimental and part of current research (6, 7). The cryopreservation of several small pieces of the immature testicular tissue and the isolation and subsequent freezing of spermatogonial stem cells (SSCs) can be performed in specialized cryobanks using stem cell freezing protocols. For a long time, techniques concerning later transplantation or *in vitro* maturation for tissues or cells of young girls as well as boys have been experimental. Besides the ethical discussion regarding the use of these biopsies and the possible dissemination of malignant cells, the techniques still need to be proven in the clinical routine for childhood cancer survivors (8–10). There are only a few case reports of ovarian tissue cryopreservation (OTC) during childhood, transplantation in the adolescent female and subsequent successful pregnancies and deliveries (11, 12). OTC in the adult female and transplantation is an already accepted option (13–16). *In vitro* maturation of immature oocytes from prepubertal females harvested during the OTC procedure showed a lower maturation potential compared with oocytes from adult females (17). Recently, the first deliveries were reported after *in vitro* maturation of oocytes collected at the time of OTC and subsequent vitrification (18). The transplantation techniques for male childhood cancer survivors are still more experimental (19–21). Some of the options include grafting or injection of the thawed SSCs in the remaining testis, *in vitro* models like testicular organoids, or *in vitro* growth and differentiation (22, 23). Full spermatogenesis after grafting is demonstrated in several animal models (24–27). The success of grafting and other methods still seems to be linked to the maturation state of the donor testis (prepubertal, pubertal, or adolescent) even keeping in mind that animal models have a shorter life span and shorter time of puberty. Mimicking the puberty of the male *in vitro* is still challenging (23, 28).

## Options of fertility preservation during and after puberty

Besides the possibilities for prepubertal children (OTC and SSC, respectively), there are more options in the older child, especially for boys. Young males during and after puberty are more likely able to ejaculate. Motile sperm of those samples can be frozen according to state-of-the-art protocols of the IVF or andrology/urology lab using slow freezing or vitrification before the gonadotoxic therapy starts (29–31). If the ejaculated sample does not contain enough sperm, the child can be counseled for testicular tissue cryopreservation (4). Young female patients during puberty under the age of 18 have a contraindication for controlled ovarian stimulation due to their age because the medication is not licensed for this age group in Germany. Young females can be counseled for an off-label use to combine both techniques, OTC and freezing of mature oocytes. Recent approaches even combine OTC and *in vitro* maturation of immature oocytes (32, 33).

As a second option, gonadotropin-releasing hormone agonists (GnRHa) can be offered to female patients after puberty (2). Unfortunately, the results of the meta-analysis of the application of GnRHa are conflicting because different endpoints were examined, e.g., the prevalence for premature ovarian insufficiency, the duration of amenorrhea, or pregnancy rate (2).

## Counseling young patients with cancer

In Germany, counseling of a young patient with cancer and/or before a gonadotoxic therapy starts is considered necessary for the patient. Therefore, patients can make a comprehensive decision as part of the overall therapy strategy. Hence, the obligation to offer advice is described in the national guidelines (2). It also needs to be taken into account that children are not legally allowed to make a decision, which means that the child’s parents need to be counseled along with the child. The aim of counseling intervention is to support the decision-making process and reduce possible decisional conflicts and anxiety and also to depict a strategy for the future. The decision to undergo fertility preservation may be affected by a multitude of psychosocial factors. Most patients who are referred for fertility preservation counseling are in the early stages of coping with their cancer diagnosis. The patients may be struggling with their mortality, future recurrences, and illness-related sequelae (34–36). Additionally, the preexisting anxiety regarding the illness itself can be intensified by the time sensitivity of the decision (37). Another burden in counseling is the financial burden to the child or the child’s family when pursuing fertility preservation because most insurance policies do not cover the treatment costs. Besides all these issues, it is widely accepted that patients benefit from counseling depicting choices and, therefore, making them visible and relevant (38, 39).

## The burden of costs for fertility preservation

Health insurance companies unfortunately do not cover the costs for fertility preservation in the pediatric population in general. We need to consider that the time of storage of the OT or SST might be up to 30 years until the former children want to start a family. Regulations concerning cost coverage of fertility preservation vary worldwide and change constantly. Since July 2021 in Germany, the costs for MII freezing in patients >18 years old are covered by health insurance when, e.g., the mammary cancer is not hormone receptor–positive. Oocyte freezing has been covered in the state of New York for medical reasons since March 2021. Freezing of ejaculated sperm or TESE biopsies is already covered in pubertal male patients. The costs of cryopreservation in female patients <18 years old and in pre/pubertal male patients can be supported by foundations or clinical studies if applicable. Some cryobanks offer freezing of SSC for free as long as the procedure remains within an experimental status.

## The network Fertiprotekt

In German-speaking countries, the network Fertiprotekt (https://fertiprotekt.com/) collects national data for cryopreservation of cells and tissue of female patients. The network was founded in 2006, and more than 150 centers located at universities or private settings are voluntary members by now. The data are published annually as part of the national IVF registry (https://www.deutsches-ivf-register.de/). In 2020, more than 1500 female patients were counseled regarding their options for fertility preservation (https://www.deutsches-ivf-register.de/perch/resources/dirjb2020en.pdf) and about 1000 decided to perform some kind of preservation option, including GnRHa. Approximately 60 young female patients under 15 years of age were counseled and about 160 young females between 15 and 20 years old. In 2020 in total, 327 OTCs were performed in German-speaking countries.

## Own data

Getting more into detail for the female pediatric patient, we now present our own data for this particular group. Between 2018 and May 2022, OTCs of 104 girls with a mean age of 14 years (range 1–17 years) were frozen at the UniCareD Cryobank in Düsseldorf. These girls and their parents were counseled in different centers, universities, and hospitals in their pediatric oncology or hematology departments within Germany. The surgery was performed in either the pediatric surgery unit of the university hospital in Düsseldorf or a surgery unit near their hometown with overnight shipping in specialized boxes. All the referring centers have signed cooperation contracts. As soon as the girl and her parents agree to the option of cryopreserving OT, the centers announce the surgery before the start of the gonadotoxic treatment. A special transport box with cooling packs that keep the temperature between 4°C and 8°C for 24 h is offered from the UniCareD within 24 h after announcement. The overnight shipping is performed between 4°C and 8°C in an organ transport medium (Custodiol ^®^, Dr. Franz Köhler Chemie GmbH, Bensheim, Germany). According to our checklist, the centers keep the cooling packs and the tube filled with Custodiol in a fridge on-site. All surgeons are advised to observe a further piece of the ovary for possible metastasis in the pathology unit on-site. As part of our setup, we also assessed 3 × 2 mm ovarian biopsies before cryopreservation to assume a vitality score (follicle count) after enzymatic digest with collagenase and fluorescent staining with calcein AM (both Merck KGaA, Darmstadt, Germany). This follicle count ranged from 1 to 1000 follicles per 3 × 2 mm biopsies with a mean of 202 (median 160). This follicle count can be used to plan the number of cortex pieces to transplant later. The cryopreservation process in the UniCareD starts with the preparation of the cortex within a class A hood on a cool plate. The cortex pieces are frozen according to a slow freezing protocol with automatic seeding in multipurpose handling medium (MHM, Fujifilm Irvine Scientific, Santa Ana, CA, USA) supplemented with DMSO as a cryoprotectant. Long-term storage of the samples is performed in the gas phase in a liquid nitrogen tank. Focusing on the pathologies, 1/3 of these patients suffered from a Hodgkin lymphoma, 1/10 from Ewing sarcoma, and <1/10 from osteosarcoma and ß-Thalassemia during initial diagnosis. Two patients were counseled with a recurrent malignancy. All physicians and the patients were informed about the results of the vitality test, the number of pieces of the ovarian cortex frozen (ranging between 3 and 10), and the long-term storage of the samples.

## Discussion

The number of cancer survivors who suffer from either childhood or adolescent cancer is rising due to the latest developments in chemotherapies by using new schemes or targeted therapies. Every patient of reproductive age and every child should have free access to an oncofertility consultation prior to gonadotoxic therapy. There should be no financial burden or lack of a time slot. Centers specialized in the counseling and treatment of patients at risk for fertility loss can try to optimize their work, e.g., with a specialized team for fertility preservation, dedicated phone number or mail contacts, standard operating procedures on-site, and networking at their location or nationwide (e.g., Fertiprotekt). Propagating all the information about the options for fertility preservation on the website of the specialized centers will improve the referral of juvenile as well as adolescent patients at risk. As it stands, the referral rate to an oncofertility unit in Germany for female patients diagnosed with cancer is below 10%, unfortunately (40). Health care providers and physicians of different disciplines can be offered more information, and legal aspects can change, e.g., the cost coverage of MII oocytes for medical reasons in different countries worldwide. Acquiring data on the incidence of cancer, the performance of fertility preservation techniques, freezing conditions, storage, and the later use of the tissue or cells is necessary to improve the outcome for the patients, especially for childhood cancer survivors with partially experimental techniques. One of these aims is to fully grow and mature oocytes *in vitro* from strips of ovarian tissue for patients at high risk of reintroduction of malignant cells by retransplantation (41, 42). Furthermore, the outcome for female patients could be optimized by maturation of immature oocytes aspirated during the endoscopic biopsy of ovarian tissue (43). Thawing and transplantation techniques for male patients need to be refined, but in our opinion, research is progressing, and male grafting or *in vitro* differentiation might possibly be realized in 5 or 10 years.

## Data availability statement

The original contributions presented in the study are included in the article/supplementary material. Further inquiries can be directed to the corresponding author.

## Author contributions

All authors listed have made a substantial, direct, and intellectual contribution to the work, and approved it for publication.

## Conflict of interest

The authors declare that the research was conducted in the absence of any commercial or financial relationships that could be construed as a potential conflict of interest.

## Publisher’s note

All claims expressed in this article are solely those of the authors and do not necessarily represent those of their affiliated organizations, or those of the publisher, the editors and the reviewers. Any product that may be evaluated in this article, or claim that may be made by its manufacturer, is not guaranteed or endorsed by the publisher.
